# Mental Health Profiles in a Sample of Moroccan High School Students: Comparison Before and During the COVID-19 Pandemic

**DOI:** 10.3389/fpsyt.2021.752539

**Published:** 2022-02-21

**Authors:** Abdennour El Mzadi, Btissame Zouini, Nóra Kerekes, Meftaha Senhaji

**Affiliations:** ^1^Department of Biology, Faculty of Sciences, Abdelmalek Essaadi University, Tetouan, Morocco; ^2^Department of Health Sciences, University West, Trollhättan, Sweden

**Keywords:** COVID-19 pandemic, Brief Symptom Inventory (BSI), gender, psychological distress, physical and/or psychological abuse, physical activity

## Abstract

**Background:**

Adolescent high school students may be particularly susceptible to suffering from the effect of isolation, physical distancing restrictions, and school closures imposed during the COVID-19 (Corona Virus Disease 2019) pandemic. Given the biological and psychological changes that occur during this period of development, adolescents' experiences of these pandemic measures could significantly threaten their mental health and cause long-term consequences.

**Aim:**

The main objectives of the study were to determine the impact of confinement because of the COVID-19 pandemic restrictions on the psychological distress of Moroccan adolescents and identify the risk and protective factors that could influence their mental health.

**Methods:**

The participants in this study were Moroccan high school students who were recruited at two different times—before the COVID-19 pandemic (350 students, mean age: 16.55 years; 53.71% female; data collected in 2014/2015) and after the announcement of the pandemic (457 students, mean age: 16.84; 64.1% female; data collected in 2020). Students responded to an anonymous survey that included several validated instruments, such as the Brief Symptom Inventory and the Godin-Shephard Leisure-Time Physical Activity questionary, and elicited information about the students' psychosocial environment, gender, and age. The scores on the Brief Symptom Inventory dimensions from the pre-pandemic period and during 2020 were compared. A comparison between the scores of the two genders of the 2020 sample was also carried out. In addition, binary regression analysis was performed to predict the associations between gender, frequency of physical activity, the presence of the number of negative psychosocial factors, and those dimensions of the Brief Symptom Inventory that significantly changed between the samples.

**Results:**

Female students reported higher psychological distress than male students in both data collection periods. During the COVID-19 pandemic, students scored significantly (*p* < 0.001) higher in depression and paranoid ideation, and they scored significantly (*p* = 0.01) lower in hostility and anxiety compared with the pre-pandemic period. Female gender and the experience of physical or psychological abuse significantly increased the risk of reporting higher scores in depression and paranoid ideation symptoms during 2020. Moderate and frequent physical activities were significantly and negatively associated with depression (*p* = 0.003 and *p* = 0.004; respectively).

**Conclusions:**

This study confirms the stressful impact of the COVID-19 pandemic on Moroccan high school students, who reported more symptoms of depression and paranoid ideation compared with the pre-COVID-19 period. Female students reported higher psychological distress than male students did. The experience of physical /psychological abuse during the pandemic worsened mental health, while moderate/frequent physical activity improved it.

## Introduction

Coronavirus disease of 2019 (COVID-19) has caused a public health emergency that has raised concerns internationally. On March 11, 2020, the World Health Organization (WHO) declared that the spread of COVID-19 had reached the status of a global pandemic (1). To slow the spread of the pandemic, many governments introduced measures that reduce physical contact by enforcing social distancing (2). In this context, on March 24, 2020, the Moroccan government decided to impose a national lockdown to limit the spread of the disease by closing most public establishments, preventing all social and entertainment activities outside the home, and restricting the movement of people by imposing exceptional authorization of one member of each family (3).

The confinement is a difficult psychological and social experience for most people; it requires physical and social distancing, including separation from family and friends, as well as frustration resulting from the commitment to sit at home. The government restrictions could have disastrous consequences for mental health (4, 5). In fact, mental health issues resulting from the COVID-19 outbreak are common in various subpopulations, including confirmed patients (6), frontline professionals healthcare (7), and elderly persons (8). Importantly, social distancing may increase mental health problems in adolescents, who are already more vulnerable to developing mental health problems compared with adults (1, 9, 10) because of adolescents' increased desire for autonomy and connection with peers (11).

The United Nations Sustainable Development Group (UNSDG) shows that the COVID-19 pandemic has created the biggest upheaval in education systems history, affecting nearly 1.6 billion students in more than 190 countries (12). Closings of schools and other educational institutions affected 94% of the world's student population, a proportion that has risen to as high as 99% in low- and middle-income countries (12).

Morocco, among many countries, imposed a complete closure of all schools at all educational levels (13), resulting in a general isolation of students at their homes for a period of around 4 months (from March 2020 to July 2020). As a result, face-to-face studies were replaced by distance learning, leisure activities outside the home stopped, and all organized sports and collective physical activities were prohibited. Indeed, most adolescents adopted sedentary behaviors at home; therefore, a reduction in physical activity was observed (14). Previous research has shown that decreased physical activity is associated with worsened mental health profiles (15, 16), whereas lower levels of depression are associated with more time spent engaged in physical activity, including team sports, gym exercises, and walking outdoors (17–19).

Loades et al. (20) found that social isolation and loneliness affect young people; the impact of the COVID-19 pandemic's restrictions on health is potentially significant and of particular concern regarding the mental health of children and adolescents (21). Unfortunately, social distancing measures can lead to social isolation in an abusive household, and that abuse is especially likely to be exacerbated during this time of financial/social instability, fear of infection, boredom, and frustration (22). Previous research has shown that adolescent abuse may be associated with post-traumatic stress disorder (23) and low self-esteem (24), which are strongly linked to internalized and externalized behaviors and negative effects such as depression, anxiety, hostility, somatization, and psychoticism (25). In addition, parental substance use problems may affect the mental health of children. Recent studies caried out on Moroccan and Swedish adolescents show that adolescents who have at least one parent with substance use problems (drugs and/or alcohol) reported more mental health problems (25, 26).

In addition, adolescence is often seen as a period of challenges that can lead to mental health problems (27, 28), especially that more than 50% of mental disorders in adults occur before the age of 18 (29). For example, in Morocco one in five adolescents suffers from a mental disorder; in half of these cases, the age of onset was 14 years (30, 31). According to the results of two studies carried out in Tunisia and Egypt, 18.7% of Tunisian adolescents aged 15 to 17 suffer from anxiety and 5.2% from depression (32), whereas 25.5% of Egyptian adolescents suffer from anxiety disorders (33). The prevalence of psychological distress among adolescents in sub-Saharan Africa countries is close to that found in North African ones, this prevalence was 23.0% in Tanzania (34), 24.2% among urban out-of-school adolescents in Nigeria (35), 15.7% in Zambia (36). In Tanzania, the prevalence of single psychological distress in adolescents was 20.6% while that of multiple psychological distress was 10.3% (37).

However, to date, very few studies in Morocco and African countries have focused on adolescent mental health during the COVID-19 pandemic. Since the mental health of today's adolescents determines the stability and security of tomorrow's society, it is essential to consider the psychological well-being of children and adolescents during crises. This consideration is particularly vital because young people are more likely to experience the long-term consequences of problems related to their mental health profile (38).

There is a scarcity of studies focused on adolescent mental health during the COVID-19 pandemic in Morocco. Moreover, previous studies (25, 26) conducted as part of the project to which this study belongs “Mental and Somatic Health without borders” (MeSHe) project (39) have shown an association between negative psychosocial factors, such as parental alcohol or drug use problems and experiences of physical or psychological abuse, and adolescent mental health profiles. Therefore, the present study aims to examine how the mental health of Moroccan adolescents has changed during the COVID-19 pandemic, to determine the impact of the negative psychosocial factors mentioned above on mental health profiles, and to determine the effect of physical activity, which is assumed to be a protective factor.

## Method

### Study Population

This study was carried out within the framework of the “Mental and Somatic Health without borders” (MeSHe) project (39). The MeSHe survey, founded by the project leader and co-author (NK), focuses on somatic and mental health profiles coupled with substance use and aggressive antisocial behaviors in adolescents in an international context.

The participants in the present study were Moroccan high school students recruited in two different periods:

- 2014/15, before the COVID-19 pandemic
- 2020, during the COVID-19 pandemic

#### The 2014/15 Sample

A detailed description of this population can be read in the study by Zouini et al. (25). Briefly, using convenience sampling, 375 high school students (170 boys and 205 girls) were selected from classes of four high schools located in different areas in Tetouan, Morocco, during the academic year of 2014/15 and participated in this study. The high schools had a total of 97 10th, 11th and 12th grade classes. Two classes from each grade and from each school were conveniently selected to participate in the study. In these 24 classes, there were 876 students of which 375 (43%) completed the survey. The age range of the participants was 15–19 [mean (*M*) = 16.56, standard deviation (*SD*) = 1.04] years.

#### The 2020 Sample

From September 2020 to February 2021, 616 students aged 15–19 years completed the MeSHe survey from Morocco. During the pandemic, contact with high school students was made in part via social media (Facebook and Instagram) and via high schools by sharing the online questionnaire link with high school principals who in turn share it with students using the website or official pages of the establishment, and groups of classes created on WhatsApp. A substantial proportion (81.84%) of the data originated from Tetouan city.

In the data files for the two different samples, students not reporting their gender or reporting genders other than male or female, those who had more than 5% missing data concerning the measure of mental health [Brief Symptom Inventory (BSI); see below], and those who gave no answer on negative psychosocial factors or leisure-time physical activity were excluded. The final samples for which data were analyzed comprised 350 students (162 boys, 188 girls) with a mean age of 16.55 (*SD* = 0.96) from the 2014/15 data file and 457 high school students (164 boys, 293 girls) with a mean age of 16.84 (*SD* = 1.22) from the 2020 data file.

### Measures

The MeSHe project (39) assesses information from high school students by means of a standardized, self-reported anonymous survey (the MeSHe survey). Alongside background information, such as age, gender, and the presence of negative psychosocial factors [parental alcohol use problems (PAP), parental drug use problems (PDP), experiences of physical abuse (PHA), experiences of psychological abuse (PSA)], the MeSHe survey consists of validated questionnaires in which young people rate their mental and physical health; aggressive, antisocial, and self-harm behaviors; and substance use habits, as well as answering questions related to their physical activity and personality. This study focuses on the responses considering students' mental health (captured by the BSI), physical activity (measured by the Godin–Shephard Leisure-Time Physical Activity Questionnaire) and the existence of any negative psychosocial factors reported by the adolescents.

The BSI is a brief form of the Symptom Checklist Revised (SCL-90-R), a self-reporting inventory developed to measure respondents' degree of psychological distress (40). The BSI has been translated into several languages, including Arabic (41). The responding adolescents rated the general influence of each item during the past year on their wellbeing.

The BSI contains 53 items, each of which is rated on a 5-point Likert scale ranging from 0 (“not at all”) to 4 (“extremely”). Nine primary symptom dimensions of distress are assessed in the BSI—namely, somatization (SOM), obsessive-compulsiveness (OBS), interpersonal sensitivity (INS), depression (DEP), anxiety (ANX), hostility (HOS), phobic anxiety (PHOB), paranoid ideation (PAR), and psychoticism (PSY). In addition to the nine symptom dimensions, three global indices of distress—the Global Severity Index (GSI), Positive Symptom Distress Index, and Positive Symptom Total—can be calculated (40). In this study, only the GSI, an indicator of the current overall level of distress, was calculated. Here, the validated Arabic (Syrian) version of the BSI (41) was used, with acceptable (0.67) to good (0.88) internal reliability in all the primary symptom dimensions.

The Godin–Shephard Leisure-Time Physical Activity Questionnaire (42) is a self-rated instrument that measures usual leisure-time physical activity during no specific timeframe. The subject simply estimates a usual 7-day period. We calculated the total leisure-time activity score in metabolic equivalents (METs). The reported weekly frequencies of strenuous, moderate, and mild activities are multiplied by nine (strenuous), five (moderate), or three (mild) to calculate the health contribution score using the following formula: (frequency of strenuous physical activity ^*^ 9 METs) + (frequency of moderate physical activity ^*^ 5 METs) + (frequency of mild physical activity ^*^ 3 METs) (42). The health contribution score is subdivided into three categories as follows: ≥24 units (~14 kcal/kg/week or more), active (having substantial health benefit); 14–23 units (between 7 and 13.9 kcal/kg/ week), moderately active (some health benefit); and less than 14 units (less than 7 kcal/kg/week or more), insufficiently active (low health benefits) (43). This inventory was validated by Godin and Shephard (42) in an adult population showing significant correlations between points assessed and actual percentage of body fat, and maximal aerobic power, and it has also been validated in the adolescent population (44) and was equally reliable in girls and boys indicating that it may be useful for comparing physical activity levels between groups and examining changes in activity levels of groups over time (44).

### Ethical Considerations

The MeSHe survey was designed in accordance with the Helsinki Declaration (45). The use of the survey in Morocco was approved by the Regional Directorate of the Ministry of National Education in Tetouan (with registration number 85) and by the Faculty of Science, University Abdelmalek Essaadi, during the period leading up to the announcement of the COVID-19 pandemic. The directorate is responsible for managing and directing all matters concerning students from primary to high school education in Tetouan province. The survey was voluntary and anonymous, and it required written consent from the respondent. For online data collection after the announcement of the COVID-19 pandemic, online informed consent was obtained from all subjects.

### Statistical Analyses

The IBM™ Statistical Package for the Social Sciences (SPSS) version 26 software program was used to analyze the data. The independent *t-*test was used to compare the BSI scores of male and female students and those of the 2014/15 and 2020 samples. The strength of the statistically significant comparison was evaluated using Cohen's d effect size with the following d values: 0.2 represents a “small” effect, 0.5 a “medium” effect, and 0.8 a “large” effect (46).

We replaced the missing values in the BSI items with the means by gender if a respondent had <5% missing data (under three items). Otherwise, the response was eliminated.

The method of classification by clusters was used to transform each of the continuous variables “Paranoid Ideation score” and “Depression score” into two clusters characterized by a good quality of cohesion and separation, with average silhouettes of classification 0.7 and 0.6, respectively:

- PAR score:
  ➢ Cluster 1 (31.5%): Group 1: low levels of PAR symptoms (group mean =0.69);
  ➢ Cluster 2 (68.5%): Group 2: high levels of PAR symptoms (group mean =2.32).
- DEP score:
  ➢ Cluster 1 (48.4%): Group 1: low levels of DEP symptoms (group mean=0.71);
  ➢ Cluster 2 (51.6%): Group 2: high levels of DEP symptoms (group mean =2.29).

Two nominal variables were then created. In addition, binary regression analysis was used to predict the association between gender, the frequency of physical activity, the presence of any negative psychosocial factors—specifically, PDP, PAP, PHA, PSA—and the fact of scoring highly in DEP and PAR symptoms on the BSI.

The choice of these factors involved in the regression model is based on the results of two previous studies carried out on Moroccan population (25, 47), one of these two studies is realized within the framework of the same project (MeSHe project) from which the 2014/15 sample was taken. This last study showed that negative psychosocial factors impact the mental health profile of adolescents while the other study shows that physical activity has a positive effect on psychological health.

All the analyses were two-tailed, and the significance level was defined as *p* < 0.05.

## Results

### Mental Health of Moroccan High School Students During the COVID-19 Pandemic

Table 1 summarizes the mean values for each of the nine primary symptom dimensions of the BSI and the GSI in the Moroccan student sample from 2020. Generally, the responding Moroccan female students reported higher psychological distress when compared with male students. The female students scored significantly higher on all primary symptom dimensions except for the “HOS” dimension, where no significant difference could be measured between the genders, the scores of SOM, OBS, PSY, DEP, INS, PAR differed between the two groups with a small effect size, whereas those of PHOB and ANX differed with a moderate effect size. The generally higher psychological distress level in the female students is also reflected in their significantly higher GSI scores, the GSI score differed between male and female students with a small effect size (Table 1).

**Table 1:** Self-reported psychiatric problems during the COVID-19 pandemic (*N* = 457).

**BSI subscales**	**Moroccan adolescents M (SD)**	**Male (*n* = 164) M (SD)**	**Female (*n* = 293) M (SD)**	***t*** **-Test**				**Cohen's d**
				***t***	***p***	**Mean difference**	**(95%) CI**	
SOM	1.26 (0.87)	1.02 (0.76)	1.39 (0.9)	4.55	<0.01	0.36	(0.21; 0.52)	0.42
OBS	1.76 (0.88)	1.56 (0.86)	1.88 (0.87)	3.69	<0.01	0.31	(0.15; 0.48)	0.35
PSY	1.29 (0.94)	1.16 (0.84)	1.37 (0.96)	2.42	0.02	0.22	(0.04; 0.39)	0.23
DEP	1.52 (0.96)	1.37 (0.93)	1.60 (0.97)	2.48	0.01	0.23	(0.05; 0.41)	0.24
INS	1.55 (0.93)	1.36 (0.88)	1.66 (0.95)	3.3	<0.01	0.30	(0.12; 0.47)	0.32
HOS	1.11 (0.89)	1.06 (0.80)	1.15 (0.94)	1.12	0.27	0.09	(−0.07;0.26)	0.10
PHOB	1.23 (0.89)	0.91 (0.74)	1.40 (0.92)	6.28	<0.01	0.5	(0.34; 0.65)	0.56
ANX	1.39 (0.95)	1.06 (0.81)	1.58 (0.98)	6.19	<0.01	0.53	(0.36; 0.69)	0.55
PAR	1.80 (0.96)	1.62 (0.96)	1.90 (0.94)	3.06	<0.01	0.28	(0.10; 0.46)	0.3
GSI	1.43 (0.78)	1.23 (0.71)	1.54 (0.80)	4.32	<0.01	0.31	(0.17; 0.46)	0.4

*SOM, somatization; OBS, obsessive-compulsiveness; INS, interpersonal sensitivity; DEP, depression; ANX, anxiety; HOS, hostility; PHOB, phobic anxiety; PAR, paranoid ideation; PSY, psychoticism; GSI, Global Severity Index; CI, confidence interval; COVID-19, coronavirus disease of 2019; SD, standard deviation*.

### Comparison of Moroccan High School Students' Mental Health Before and Under the COVID-19 Pandemic

Students from the 2020 data collection sample scored significantly higher in the DEP and PAR (*p* < 0.001) primary domains of BSI than those from the 2014/15 data collection sample, and the scores of these two domains differed between the two groups with a small effect size. However, students during the COVID-19 pandemic scored significantly lower in the HOS and ANX (*p* = 0.01) primary dimensions of BSI compared with the report from 2014/15, and the scores for these dimensions differed between the two groups with a negligible effect size (Table 2).

**Table 2:** Comparison of self-reported psychiatric problems between the 2020 (*N* = 457) and the 2014/15 samples (*N* = 350).

	**Before the COVID-19 pandemic**	**During the COVID-19 pandemic**	***t*** **-Test**				**Cohen's *d***
			***t***	***p***	**Mean difference**	**CI (95%)**	
SOM	1.26	1.26	−0.05	0.96	0.03	(−0.12; 0.12)	0
OBS	1.72	1.76	0.75	0.46	0.03	(−0.07; 0.17)	0.05
PSY	1.37	1.29	−1.22	0.22	−0.10	(−0.20; 0.05)	−0.09
DEP	1.22	1.52	4.61	<0.01	0.31	(0.17; 0.43)	0.32
INS	1.50	1.55	0.83	0.41	0.04	(−0.08; 0.18)	0.06
HOS	1.28	1.11	−2.74	0.01	−0.18	(−0.28; −0.05)	−0.19
PHOB	1.18	1.23	0.83	0.41	0.06	(−0.07; 0.17)	0.06
ANX	1.55	1.39	−2.51	0.01	−0.18	(−0.29; −0.04)	−0.18
PAR	1.47	1.80	5.31	<0.01	0.32	(0.21; 0.46)	0.36
GSI	1.39	1.43	0.64	0.52	0.05	(−0.07; 0.14)	0.05

*SOM, somatization; OBS, obsessive-compulsiveness; INS, interpersonal sensitivity; DEP, depression; ANX, anxiety; HOS, hostility; PHOB, phobic anxiety; PAR, paranoid ideation; PSY, psychoticism; GSI, Global Severity Index; CI, confidence interval; COVID-19, coronavirus disease of 2019; SD, standard deviation*.

Male students from the 2020 data collection sample scored significantly higher in the DEP, PAR, and INS (*p* < 0.001, *p* = 0.01 and *p* = 0.03, respectively) primary domains of BSI than those from the 2014/15 data collection sample, and the scores of these three domains differed between the two groups with a small effect size. However, during the COVID-19 pandemic, male students scored significantly lower in the ANX (*p* = 0.03) primary dimension of BSI than was reported in 2014/15, and the scores in this dimension differed between the two groups, with a small effect size (Table 3).

**Table 3:** Comparison of male students' mental health before and during the COVID-19 pandemic.

	**Before the COVID-19 pandemic**	**During the COVID-19 pandemic**	***t*** **-Test**				**Cohen's *d***
			***t***	***p***	**Mean difference**	**CI (95%)**	
SOM	0.99	1.02	0.40	0.69	0.03	(−0.13; 0.20)	0.04
OBS	1.52	1.56	0.49	0.63	0.05	(−0.14; 0.23)	0.05
PSY	1.26	1.16	−1.13	0.26	−0.10	(−0.28; 0.08)	−0.11
DEP	1.05	1.37	3.33	<0.01	0.32	(0.13; 0.51)	0.34
INS	1.16	1.36	2.12	0.03	0.20	(0.01; 0.39)	0.22
HOS	1.17	1.06	−1.32	0.19	−0.11	(−0.28; −0.05)	−0.13
PHOB	0.97	0.91	−0.71	0.48	−0.06	(−0.23; 0.11)	−0.07
ANX	1.24	1.06	−2.15	0.03	−0.18	(−0.35; −0.02)	−0.20
PAR	1.37	1.62	2.57	0.01	0.25	(0.06; 0.45)	0.28
GSI	1.19	1.23	0.41	0.68	0.03	(−0.12; 0.18)	0.04

*SOM, somatization; OBS, obsessive-compulsiveness; PSY, psychoticism; DEP, depression; INS, interpersonal sensitivity; HOS, hostility; PHOB, phobic anxiety; ANX, anxiety; PAR, Paranoid ideation; GSI, Global Severity Index; COVID-19, coronavirus disease of 2019; CI, confidence interval*.

Female students from the 2020 data collection sample scored significantly higher in the DEP and PAR (*p* = 0.01 and *p* < 0.001, respectively) primary domains of BSI than did those in the 2014/15 data collection sample, and the scores of these three domains differed between the two groups with a small effect size. However, female students during the COVID-19 pandemic scored significantly lower in the ANX and HOS (*p* < 0.01 and *p* = 0.01; respectively) primary dimensions of BSI compared with the report from 2014/15, and the scores of these two dimensions differed between the two groups with a small effect size (Table 4).

**Table 4:** Comparison of female students' mental health before and during the COVID-19 pandemic.

	**Before the COVID-19 pandemic**	**During the COVID-19 pandemic**	***t*** **-Test**				**Cohen's *d***
			***t***	***p***	**Mean difference**	**CI (95%)**	
SOM	1.49	1.39	−1.30	0.19	−0.11	(−0.27; 0.05)	−0.12
OBS	1.89	1.88	−0.18	0.85	−0.01	(−0.17; 0.14)	−0.02
PSY	1.47	1.37	−1.12	0.26	−0.10	(−0.27; 0.07)	−0.11
DEP	1.36	1.60	2.71	0.01	0.24	(0.07; 0.42)	0.26
INS	1.79	1.66	−1.48	0.14	−0.13	(−0.30; −0.04)	−0.14
HOS	1.37	1.15	−2.74	0.01	−0.22	(−0.39; −0.06)	−0.26
PHOB	1.35	1.40	0.61	0.54	0.05	(−0.11; 0.21)	0.06
ANX	1.82	1.58	−2.84	<0.01	−0.24	(−0.41; −0.07)	−0.26
PAR	1.56	1.90	4.27	<0.01	0.35	(0.19; 0.51)	0.38
GSI	1.56	1.54	−0.36	0.71	−0.02	(−0.15; 0.11)	−0.03

*SOM, somatization; OBS, obsessive-compulsiveness; PSY, psychoticism; DEP, depression; INS, interpersonal sensitivity; HOS, hostility; PHOB, phobic anxiety; ANX, anxiety; PAR, paranoid ideation; GSI, Global Severity Index; COVID-19, coronavirus disease of 2019; CI, confidence interval*.

### Risk and Protective Factors of the Significantly Increased Psychological Domains

The overall model rates for the two proposed models in binary logistic regression were very good: 66.6 and 71% for DEP and PAR symptoms, respectively. Being female significantly increased (*p* = 0.03) the risk of reporting higher scores in both the DEP and PAR primary symptom domains of BSI to almost twice the level associated with being male (Tables 5, 6). Reporting any negative psychosocial events, especially the experience of physical or psychological abuse, significantly increased a subject's risk [odds ratio (OR) = 2.25, *p* = 0.004 and OR = 3.28, *p* < 0.001, respectively] of belonging to Group 2 of the DEP clusters, which was characterized by higher scores for depression symptoms (Table 5). Reporting psychological abuse also significantly increased the risk [odds ratio (OR) = 4.25, *p* < 0.001] of belonging to Group 2 of the PAR clusters, which was characterized by higher scores for symptoms of paranoid ideation (Table 6).

**Table 5:** Association among gender, negative psychosocial factors, the frequency of physical activity, and reported higher levels of DEP symptoms assessed by logistic regression [fit measures for the model: overall model test rate (66.6%) and pseudo r-squared (0.22)].

**Logistic regression**						**AUC**
	**B**	***p***	**OR**	**95% CI for OR**		
				**Lower**	**Upper**	
Gender: girls	0.51	0.03	1.66	1.04	2.65	0.46
Comparison group: boys						
PAP: yes	0.20	0.71	1.22	0.43	3.51	0.51
Comparison group: no						
PDP: yes	0.17	0.68	1.18	0.54	2.58	0.52
Comparison group: no						
PHA: yes	0.81	<0.01	2.25	1.29	3.91	0.61
Comparison group: no						
PSA: yes	1.19	<0.01	3.28	2.00	5.37	0.65
Comparison group: no						
Frequency of physical activity		<0.01				0.38
Comparison group: Never/rarely						
Sometimes	−0.69	<0.01	0.50	0.31	0.80	
Often	−0.90	<0.01	0.40	0.22	0.75	

*PAP, adolescents reporting parental alcohol use problems; PDP, adolescents reporting parental drug use problems; PHA, adolescents reporting experiences of physical abuse; PSA, adolescents reporting experience of psychological abuse; CI, confidence interval; OR, odds ratio; AUC, area under the curve; P, probability level; B, the unstandardized regression weight*.

**Table 6:** Association among gender, negative psychosocial factors, the frequency of physical activity, and reported higher levels of PAR symptoms assessed by logistic regression [fit measures for the model: overall model test rate (71%) and pseudo r-squared (0.18)].

**Logistic regression**						**AUC**
	**B**	***p***	**OR**	**95% CI for OR**		
				**Lower**	**Upper**	
Gender: girls	0.52	0.03	1.69	1.04	2.74	0.45
Comparison group: boys						
PAP: yes	1.32	0.10	3.73	0.77	18.07	0.52
Comparison group: no						
PDP: yes	0.53	0.28	1.69	0.66	4.34	0.54
Comparison group: no						
PHA: yes	0.49	0.13	1.63	0.86	3.09	0.59
Comparison group: no						
PSA: yes	1.45	<0.01	4.25	2.31	7.82	0.65
Comparison group: no						
Frequency of physical activity		0.29				0.42
Comparison group: Never/rarely						
Sometimes	−0.27	0.30	0.77	0.46	1.26	
Often	−0.49	0.13	0.61	0.33	1.15	

*PAP, adolescents reporting parental alcohol use problems; PDP, adolescents reporting parental drug use problems; PHA, adolescents reporting experiences of physical abuse; PSA, adolescents reporting experiences of psychological abuse; CI, confidence interval; OR, odds ratio; AUC, area under the curve; P, probability level; B, the unstandardized regression weight*.

The binary logistic regression results show that the frequency of physical activity is significantly (*p* = 0.002) negatively associated with high scores in the DEP primary domain of the BSI (Table 5). In fact, we found that moderate or frequent exercise was associated with a much more significant decrease in the risk of belonging to Group 2 of the DEP clusters (*p* = 0.003 and *p* = 0.004, respectively) than was the absence or scarcity of physical activity.

## Discussion

### Mental Health of Moroccan High School Students During the COVID-19 Pandemic

Our results show that female students reported significantly more symptoms on all the BSI primary domains, except for HOS, compared with male students. This finding is in accordance with the results of other studies carried out before the appearance of the COVID-19 pandemic (25, 26, 48, 49) and even with those performed during the COVID-19 pandemic (50–53), which all confirm that mental health problems have a feminine trend associated with the fact that girls more often experienced negative aspects of social interactions, performance and responsibility (54), higher level to react to stressful life events (24), low self-esteem rate (55) in comparison to boys, in addition to hormonal, biological and developmental differences between the two genders (56–59).

The increased distress level in female high school students generally could be associated with differences between girls and boys from a hormonal perspective; Albert (56) found that hormonal changes in girls, especially during puberty, can be a trigger for depression, and thus, mental health problems. In addition to hormonal differences, biological and developmental differences may also be implicated. During adolescence, the amygdala and hippocampal volume changes differ according to gender; the amygdala volume increases significantly only in boys, whereas the hippocampal volume increases significantly only in girls (57). These cerebral areas have also been associated with such disorders as depression and anxiety; therefore, the differences found at the developmental scale may be associated with the distinct gender differences in mental health profile (58, 59).

Girls and boys do not react to stressful life events in the same way. Girls experience higher levels of episodic stress and are more responsive to these stressors, which increases their likelihood of having a high level of psychological distress compared with boys (54, 60–63). Previous studies have shown that girls exposed to a stressful event, such as stress-related school, an experience of physical or psychological abuse, parental substance use problems, parental depression, family dysfunction, and negative parenting behaviors, report more psychological distress compared with boys exposed to the same conditions (25, 26, 60, 61, 64).

### Comparison of the Mental Health Profile of Moroccan Adolescents Before and During the COVID-19 Pandemic

During social distancing, students' daily life was disrupted, and the students were isolated from their friends and routine; moreover, they were concerned about changes in local pandemic status, which could be associated with negative psychological effects, including increased stress, hostility, and anxiety (4, 65–67), especially since the crucial resources to cope with this stressful situation, such as the availability of social support, were largely absent or impaired. However, comparing the 2014/15 and the 2020 samples, we found a significant decrease in ANX in the 2020 male sample and ANX and HOS in the 2020 female sample. This result may be associated with the use of coping strategies on the part of the students in our 2020 sample to fight against their new stressors related to the COVID-19 pandemic. It is known that people use various coping methods in crisis or disaster situations (68). Kar et al. (69) suggest that “hoping for the best” is the most common way to cope, followed by “keeping busy.” Dealing with the problems faced, could also involve religious faith, trying to share feelings and to communicate with others (69).

The first indications suggest that the pandemic has changed the way media are used (70). The increase in media consumption, or consumption of specific types of content, can be seen as a positive coping strategy to decrease hostility and to cope with the stress and anxiety experienced during the initial period of social distancing (71–75). Students' use of a coping strategy, such as accessing media, can play an important role in problem-oriented or emotional adaptation, such that students can use the media as a way to keep in touch with friends and family; a source of social support (76); a tool for entertainment, monitoring the local situation, or gathering information on other pandemics; or even a way to distance themselves from the current situation, providing humor and insight. These last forms are particularly interesting because they can be considered a positive aspect of coping that helps students to believe that they have more control over the situation; as a result, they may develop fewer hostile reactions to the stressful situation (74). In addition, these last positive aspects are also positively linked to psychological wellbeing by the absence of negative affect as an indicator of subjective wellbeing, the absence of psychological symptoms as an indicator of mental health, and fulfillment as an indicator of psychological functioning in different areas of life (70).

The decrease in anxiety could also be related to the period of distribution of the questionnaires, which coincided with the passage from full to partial confinement (September 2020 to February 2021); therefore, a reduction of social distancing measures and a transition to a new study program (50% face to face and 50% distance learning) occurred during this period. This return of students to a normal life, even partially, could decrease their anxiety levels (76).

Our results show that Moroccan high school students (males and females) reported more symptoms coupled to depression and paranoid ideation during the COVID-19 pandemic compared with the before-COVID-19 period. This increase in depression symptoms reported by students from the 2020 sample could be associated with a higher likelihood of perceiving deteriorations induced by the COVID-19 pandemic in different areas of daily life, including conflict with family members; loneliness; and worry about their studies, relationships with peers, and health (20, 77–81). Indeed, Magson et al. (77) found that increased conflict with fathers was associated with more depressive symptoms; this conflict may reflect a developmental gap in the inherent desire of adolescents to connect with their peers and seek greater autonomy from their parents (82). In addition to family conflicts, increased depression symptoms may also be linked to loneliness. In fact, Loades et al. (20) found a clear association between loneliness and mental health problems in children and adolescents—specifically, depression problems; especially, the duration of loneliness appears to be a predictor of future mental health problems up to 9 years later.

Moroccan high school students belonging to the 2020 sample reported a high level of paranoid ideation symptoms compared with students from the 2014/15 sample. This increase in the inability to trust most people, feeling that others are not giving the respondent credit for their accomplishments, feeling that others will take advantage of the respondent if given the opportunity, and feeling that others are watching or talking about the respondent, in addition to the perception that others are responsible for most of the problems the respondent experiences, could be associated with physical and verbal victimization, low self-esteem and self-efficacy, interpersonal concerns, social withdrawal personality traits, and parent–child conflict (83–85). Moreover, stress is associated with self-esteem in multiple ways. Stressful events affect self-esteem, and self-esteem affects how people respond to and cope with stress (86). Stressful life events reinforce avoidant and ambivalent behaviors, as well as insecurity. As a result, the adolescents exposed to stressors, such as those related to the COVID-19 pandemic, may have low self-esteem (63, 87) and start to exhibit symptoms of paranoid ideation (88–90). Thus, the deterioration of family or parent–child relationships during the period of COVID-19 may also be related to the increase of paranoid ideation symptoms (91, 92), especially as the COVID-19 situation is considered a stressful situation for parents, who have been obliged to support their children while working from home, while at the same time, social support for parents has been lacking because of social isolation; all these factors could be responsible for an increase in parent–child conflict (10, 93–95), and consequently, enhance the chance to develop psychiatric disorders among the children (96, 97).

Our results show a significant increase in interpersonal sensitivity symptoms in males during COVID-19 compared to male adolescents from the 2014/15 sample. Given the conservative nature of Moroccan society, Moroccan males have more freedom to leave their homes than do females; consequently, males may exhibit higher interpersonal sensitivity. In fact, a study carried out by Sfendla and Hadrya (47) on a sample of the Moroccan population showed that people allowed to leave their homes are at greater risk of contracting the virus; this group exhibited high interpersonal sensitivity, which may be due to the uncertainties associated with the pandemic. In addition, quarantine conditions during the COVID-19 period cultivated a new belief in people concerning their vulnerability to harm and the fact that proximity to others is a direct threat (98); thus, some students have reported feeling that people were hostile and did not like them (99), which will gradually replace their old worldview of interpersonal relationships (100).

### Risk and Protective Factors During the COVID-19 Pandemic

Female gender is a predictor factor of depression and paranoid ideation problems. Being a girl tripled the risk of reporting increased levels of depression and paranoid ideation during the COVID-19 pandemic in Morocco. Similar results have been found by previous studies carried out before the announcement of the COVID-19 pandemic in Moroccan, Swedish, and Israeli adolescent samples (25, 26, 101); these results reinforce the conclusion that Moroccan adolescents already reported the worst mental health profile before the COVID-19 pandemic, represented by their higher depression and paranoid ideation scores compared with adolescents from other countries and confirm the feminine trait of mental health problems, as discussed above.

According to the regression model results, reporting psychological abuse during the pandemic, significantly increased the risk of reporting high levels of paranoid ideation and depression symptoms (by factors of four and three, respectively), and reporting physical abuse during the pandemic also doubled the risk of reporting high levels of depression symptoms. Physical or psychological abuse is also associated with a high level of psychological distress, as found by previous research carried out among Moroccan and Swedish high school students (30, 40). Similar to our findings, Moroccan and Swedish studies found that the high school students who reported an experience of physical or psychological abuse scored significantly higher for depression and paranoid ideation symptoms, as well as other symptoms of psychological distress, such as phobic anxiety, somatization, obsessive-compulsiveness, anxiety, psychoticism, and interpersonal sensitivity.

The experience of abuse can cause a cascade of negative consequences across multiple functional domains for children and adolescents (102–104). The experience of abuse has been linked to increased psychological distress (26, 40, 104–106) and to long-term mental health consequences, such as low self-esteem, substance abuse (107), post-traumatic stress disorder (108), suicidal behaviors and depression (24, 25, 42, 109, 110), obsessive-compulsiveness, attention deficit hyperactivity and oppositional defiant problems (111), personality disorders (112), emotional unresponsiveness, and neuroticism (113).

No association was found between parental substance use problems and depression/paranoid ideation symptoms during the COVID-19 pandemic among Moroccan adolescents; in contrast, a recent study showed that Swedish adolescents who reported parental substance use problems scored significantly higher in the PSY, PAR, DEP, and ANX dimensions of the BSI (26). In addition to the Islamic religion, which prohibits the consumption of drugs and alcohol, this non-association between parental substance use problems and the mental health problems diagnosed during the COVID-19 pandemic in the Moroccan adolescent sample can be related to the traffic restrictions required during the COVID-19 pandemic period, which contributed to reducing access to these substances, and therefore, the decrease in their consumption by parents. Consequently, there was reduced reporting of parental substance use problems from children.

A significant association between physical activity frequency and depression symptoms was found. Moderate or frequent physical activity for high school students in the 2020 sample significantly decreased their risk of reporting higher levels of depression symptoms. This result is consistent with recent publications that have studied the association of physical activity and depressive symptoms on adolescents (114–116).

During confinement, physical activity can be done at home using a variety of exercises that are safe, simple, and easy to perform. Such forms of exercise can include, but are not limited to, strengthening exercises, balance and control activities, stretching exercises, or a combination of them. Examples of home exercise include walking around the house, lifting and carrying groceries, alternating leg lunges, climbing stairs, and doing traditional strength exercises (117). In addition, adolescents may be more motivated to exercise by making exercise a family activity, using virtual classes or online videos, and creating fitness challenges. Such methods could help maintain physical function and prevent the occurrence of several mental health disorders during this critical period (117).

Recent studies have found a positive effect of physical activity on adolescent mental health, mainly on depressive symptoms, and these positive effects are linked to biological and psychosocial pathways (118, 119). The biological mechanism suggests that physical activity has a variety of interrelated changes that take place in the brain to produce a protective environment against depression; therefore, physical activity improves mental health through changes in the structural and functional levels of the brain (120, 121). Improved mental health through moderate or frequent physical activity can also be linked to the secretion of serotonin (122, 123) and endorphin (124), in relation to their antidepressant and analgesic effects, respectively. physical activity also has an impact on the regulation of cortisol (125), which reduces physiological responsiveness to stress.

### Strengths and Limitations

There are several limitations to our study that should be mentioned. Non-probability sampling, the time lag between the two samples collected before COVID-19 (2014/15) and during COVID-19 (2020), and the absence of criteria other than age for the inclusion of students during the COVID-19 period (unlike the pre-COVID period, during which high school location, high school class, and specialty programs were also considered) were limiting points in the present study. In addition, the MeSHe project has a cross-sectional conception; therefore, no conclusion about causal associations can be drawn from the data collected. In the present study, data collection was mostly limited to high schools in the city of Tetouan and its surrounding regions in northern Morocco; the results should only be generalized with caution. Although the data were collected through online self-report questionnaires, the use of self-reporting has well-known limitations—namely, self-report questionnaires depend on the respondents' ability and willingness to remember and respond honestly; thus, responses can be skewed through social desirability and recall biases (126). However, despite the constraint of availability and access to the internet for some adolescents belonging to the lower socioeconomic class, online questionnaires were the only method to assess the mental health profile of adolescents during COVID-19 isolation. Regarding the gender distribution of our study population, it should be noted that there were more female students than male ones responding to the survey in the two periods of data collection (2014/15 and 2020) this can be explained by the fact that generally girls are more likely to participate than boys in surveys (127–129). It is important to note that the survey uses previously validated instruments for data evaluation, which is one of the strengths of our study.

## Conclusion

The results of the present study reinforce previous findings on the stressful impact of the COVID-19 pandemic on Moroccan high school students. Moroccan high school students reported more symptoms of depression and paranoid ideation during the COVID-19 pandemic than they did during the pre-COVID-19 period. Female gender and negative psychosocial factors, such as the experience of physical or psychological abuse, have a significant negative impact on depression and paranoid ideation problems. The study also presents new evidence on the protective effect of moderate/frequent physical activity on disorders involving depressive and paranoid ideation symptom. Our findings have implications for several areas of intervention. First, the creation of specific centers on listening and educational guidance that work in cooperation with a specialized staff, such as psychological counselors, should be encouraged at the level of high schools to guarantee the assessment of students' mental health problems and to allow communication with their parents in a timely manner to set up an adequate effective intervention. In addition, in cooperation with teachers and psychological counselors, health authorities should identify groups at risk for early psychological intervention. Adolescent girls should also receive more attention, as they are the most affected by the repercussions of the COVID-19 pandemic. Finally, we highlight the necessity of promoting physical activity as an important preventive strategy for maintaining adolescent mental health.

## Data Availability Statement

The raw data supporting the conclusions of this article will be made available by the authors, without undue reservation.

## Ethics Statement

The studies involving human participants were reviewed and approved by the Regional Directorate of the Ministry of National Education in Tetouan (Registration Number 85). Written informed consent from the participants' legal guardian/next of kin was not required to participate in this study in accordance with the national legislation and the institutional requirements.

## Author Contributions

AM: data collection, data analysis, drafting, and revision of the manuscript. BZ: contribution to statistical analyses, responsibility for the content of the results, and critical revision of the drafted manuscript. NK: design and direction of the Mental and Somatic Health without borders (MeSHe) project, supervision of data collection, data interpretation, and critical revision of the manuscript. MS: supervision of data collection, critically important intellectual feedback on the interpretation of the results, revision of the manuscript, and monitoring of manuscript progress and revisions. All authors read and approved the final manuscript.

## Funding

Open Access Publication fee is received from University West library.

## Conflict of Interest

The authors declare that the research was conducted in the absence of any commercial or financial relationships that could be construed as a potential conflict of interest.

## Publisher's Note

All claims expressed in this article are solely those of the authors and do not necessarily represent those of their affiliated organizations, or those of the publisher, the editors and the reviewers. Any product that may be evaluated in this article, or claim that may be made by its manufacturer, is not guaranteed or endorsed by the publisher.

## Abbreviations

ANX: anxiety
AUC: area under the curve
BSI: Brief Symptom Inventory
COVID-19: coronavirus disease of 2019
DEP: depression
GSI: General Severity Index
HOS: hostility
INS: interpersonal sensitivity
M: mean
MeSHe: Mental and Somatic Health without borders project
MET: metabolic equivalent
OBS: obsessive-compulsiveness
OR: odds ratio
PAP: adolescents reporting parental alcohol use problems
PAR: paranoid ideation
PDP: adolescents reporting parental drug use problems
PHA: adolescents reporting the experiences of physical abuse
PHOB: phobic anxiety
PSA: adolescents reporting the experience of psychological abuse
PSY: Psychoticism
SCL-90-R: symptom checklist revised
SD: Standard deviation
SOM: somatization
SPSS: Statistical Package for the Social Sciences
UNSDG: United Nations Sustainable Development Group.
